# Fabrication of high-performance dual carbon Li-ion hybrid capacitor: mass balancing approach to improve the energy-power density and cycle life

**DOI:** 10.1038/s41598-020-67216-x

**Published:** 2020-07-02

**Authors:** Tandra Panja, Jon Ajuria, Noel Díez, Dhrubajyoti Bhattacharjya, Eider Goikolea, Daniel Carriazo

**Affiliations:** 1 Centre for Cooperative Research on Alternative Energies (CIC energiGUNE), Basque Research and Technology Alliance (BRTA), Alava Technology Park, Albert Einstein 48, 01510 Vitoria-Gasteiz, Spain; 2 Universidad del País Vasco, UPV/EHU, 48080 Bilbao, Spain; 3 Instituto de Ciencia y Tecnología del Carbono, INCAR-CSIC. Francisco Pintado Fe, 26, 33011 Oviedo, Spain; 4 IKERBASQUE, Basque Foundation for Science, 48013 Bilbao, Spain

**Keywords:** Energy storage, Materials for energy and catalysis

## Abstract

Most lithium-ion capacitor (LIC) devices include graphite or non-porous hard carbon as negative electrode often failing when demanding high energy at high power densities. Herein, we introduce a new LIC formed by the assembly of polymer derived hollow carbon spheres (HCS) and a superactivated carbon (AC), as negative and positive electrodes, respectively. The hollow microstructure of HCS and the ultra large specific surface area of AC maximize lithium insertion/diffusion and ions adsorption in each of the electrodes, leading to individual remarkable capacity values and rate performances. To optimize the performance of the LIC not only in terms of energy and power densities but also from a stability point of view, a rigorous mass balance study is also performed. Optimized LIC, using a 2:1 negative to positive electrode mass ratio, shows very good reversibility within the operative voltage region of 1.5–4.2 V and it is able to deliver a specific cell capacity of 28 mA h^−1^ even at a high current density of 10 A g^−1^. This leads to an energy density of 68 W h kg^−1^ at an extreme power density of 30 kW kg^−1^. Moreover, this LIC device shows an outstanding cyclability, retaining more than 92% of the initial capacity after 35,000 charge–discharge cycles.

## Introduction

The search for more powerful energy storage devices has been intensified in recent years due to the increasing energy demand from modern human activity. Thus, both the research and the industrial communities are facing the challenges to develop high power/energy sources for the fast-growing market of electric vehicles, aerospace and next generation portable electronics. Amongst the different energy storage systems, lithium-ion batteries (LIBs) and supercapacitors (SCs) are the preferred energy sources for high energy or high-power applications, respectively. The main advantages of LIBs over SCs are their broad operating potential window, their higher energy density (∼200 W h kg^−1^ *vs*. < 10 W h kg^−1^ for SCs)^1–4^. On the other hand, SCs are able to supply much higher power densities and have an extended cycle life (over 10^6^ cycles). Industrially manufactured LIBs and SCs still show their limitations in certain areas of application demanding both high-power and high-energy.

Hybrid electrochemical capacitors (HECs), which combine a battery-type negative electrode with a capacitive positive electrode, have recently attracted huge scientific and industrial interest since they can provide high energy densities at high power. HECs based on different metal-ions such as Li^+^, Na^+^ or K^+^ have been proposed until date^5–12^. In particular, in lithium-ion capacitors (LICs) the intercalation/deintercalation of Li^+^ occurs in the anode side as in a LIB, whilst the adsorption/desorption of the counter ion (typically PF_6_^−^) takes place at the surface of the positive electrode as in an electrical double layer capacitor (EDLC)^5,6^. Different LIC systems (“Dual carbon LICs”) combining a high surface area activated carbon as the positive electrode with a Li-ion intercalating carbon (graphite, hard carbons or soft carbons) as the negative electrode have been described in the literature showing an energy storage capacity almost five times higher than that of EDLCs, maintaining good response at high power demand and stability upon long cycling.^6,9,10-12^. Another advantage with respect to conventional EDLC capacitors is that, due to the asymmetric combination of anode and cathode, the LIC devices suffer from a much lower self-discharge, similarly to Li-ion batteries^7,11^.

Regarding the negative electrode, hard carbons have shown promising results even doubling the theoretical capacity of graphite. Their disordered structure containing graphite-like domains with a low degree of crystallinity enables the use of more space for the Li^+^ ion storage in the cavities and micropores along with intercalation^10,12^. Different nanostructured carbon materials, namely carbon nanosheets, nanospheres, carbon nanopipes or carbon nanofibers among others have been recently investigated as anodes for LICs. Tuning the microstructure of the carbons at the nanoscale brought about significant improvements in terms of structural stability, transport kinetics, cyclability, and coulombic efficiency^9,12–14^. The morphology of the hollow carbon spheres results particularly convenient since they provide electrolyte reservoirs and fasten Li^+^ intercalation/deintercalation processes through the thin carbon walls^15,16^. Additionally, their ample inner space can buffer the volume changes undergone during the charge/discharge processes, thus improving the mechanical stability of the electrode^16,17^.

As the positive electrode, activated carbons are preferentially chosen due to their large specific surface areas and open porosity, which allows fast ionic transport to the whole surface of the electrode^6,18^. In a previous work, we have introduced a novel and straightforward synthetic route for the preparation of ultra-high specific surface area activated carbons. This synthesis strategy, consisting on a facile one-step process in which polymerization, carbonization and chemical activation of the carbon precursors occur all at once and yields carbons with specific surface areas slightly above 3000 m^2^ g^−1^ and a hierarchical micro-mesoporous structure. Both their suitable porous structure and easy preparation make them a suitable choice for the positive electrode material in LIC systems^19^.

The performance of hybrid supercapacitors can be improved through the optimization of the mass balance between the positive and negative electrodes^20^. Thus, different mass balances translate into different working potential spans and, therefore, a different degree of utilization of each electrode, which can be used to maximize the energy density of the device. Indeed, most of the scientific reports focus on the best-obtained energy/power results, not paying much attention to safety and stability.

In this report, we present a facile synthetic route towards hollow carbon spheres by the pyrolysis of nitrogen containing monomers. This material was coupled in a full cell with our home-made superactivated carbon as the positive electrode. Optimization of the electrodes mass balance, within an operative potential window of 1.5–4.2 V, was also investigated.

## Results and Discussions

### Physicochemical characterization

**Figure 1:**
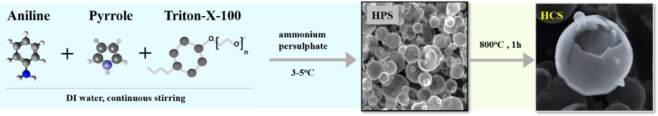
Schematic diagram of the synthesis process of hollow polymer spheres (HPS) and hollow carbon spheres (HCS).

The schematic diagram included in Fig. 1 summarizes the approach followed for the preparation of the hollow carbon spheres that will serve as negative electrode in this study. First, polymeric hollow microspheres were prepared by a simple strategy that involves the interfacial co-polymerization of aniline and pyrrole in the presence of Triton X-100^17,21^. Due to their different hydrophobicity, the molecules of aniline mainly sit at the outer layer of the micelle-water interface, whereas the more hydrophobic pyrrole molecules tend to diffuse towards the inner wall of the micellar core.

**Figure 2:**
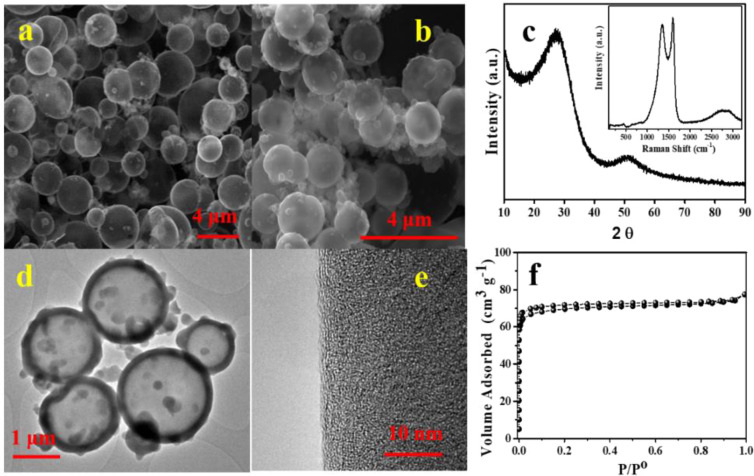
(**a**) SEM images of HPS and (**b**) HCS obtained after the pyrolysis of HPS, (**c**) XRD pattern recorded for HCS (inset: Raman spectrum registered for HCS), (**d**) low magnification TEM image of HCS and (**e**) high magnification TEM image of HCS outer surface, (**f**) N_2_ adsorption-desorption isotherms registered for HCS.

Polymerization leads to the formation of hollow polymeric spheres (HPS) and its subsequent carbonization under inert atmosphere yields hollow carbon spheres (HCS). The HCS maintain the pristine microstructure of the HPS but undergo a slight shrinkage of their size (Fig. 2a,b).

The thickness of the carbon walls was of *ca*. 110 nm (Fig. 2d). The XRD pattern of the carbonized sample (Fig. 2c) shows two low intensity and broad X-ray diffraction peaks at ~26° and ~50°, which correspond to the (002) and (100) planes characteristic of disordered carbons with a low degree of graphitization. The Raman spectrum (Fig. 2c, inset) shows two predominant bands at ~1356 cm^−1^ and ~1594 cm^−1^, which reflect the defects in the carbon lattice (D-band) and the stretching vibration in C-C bonds (G-band), respectively. Additionally, two broad and very low intense peaks can be identified in the 2500–3000 cm^−1^ region, that are ascribed to the G´ stretching mode. Defects in the forms of edges and surface imperfections like defects, cracks, cavities, and active sites act as catalytic sites, which can be active for formation of solid-electrolyte interface (SEI) layer as well as lithiation-delithiation process in the negative electrode of LIC cell^22^. The high-resolution TEM images (Fig. 2e) evidenced the presence of micropores in the carbon shells. To get additional information about the textural features of these carbon spheres, nitrogen gas adsorption-desorption measurements were carried out. The N_2_ adsorption-desorption isotherm registered for HCS (Fig. 2f) shows a profile in between types I and IV according to IUPAC classification, with a H4 hysteresis loop^23^. The large adsorption of nitrogen at low relative pressures confirmed the microporous nature of the material. Due to the presence of a large amount of micropores, the BET specific surface area calculated for this material was 282 m^2^ g^−1^. Since the monomers used for the preparation of the HCS contain nitrogen we have also performed the elemental analysis of the HCS carbon using Inductively Coupled Plasma Mass Spectrometry (ICP-MS) to determine the nitrogen content in this anode material. The analysis showed that HCS has a high nitrogen content of 9.1 wt.% in its carbon framework. It is well-known that the incorporation of nitrogen-containing groups in the carbon network not only improves the electronic and ionic conductivities but also provides active sites that enhance ion adsorption leading to an increase in capacity and rate capability^17,24^.

**Figure 3:**
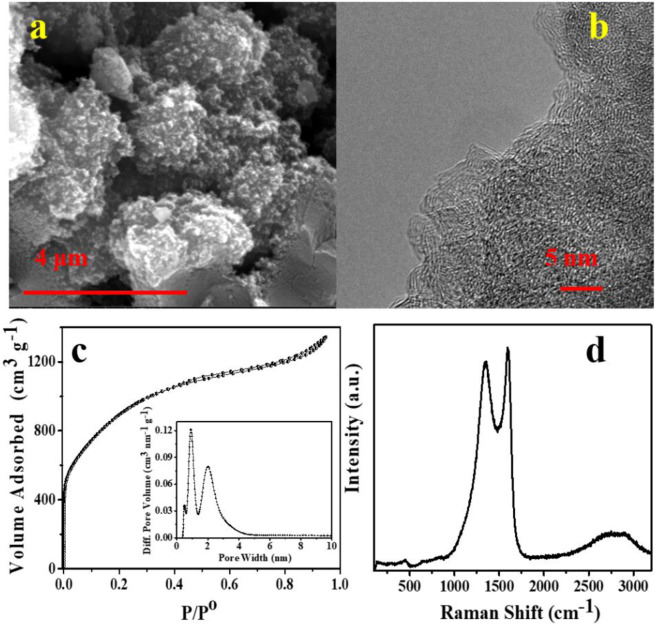
(**a**) SEM image, (**b**) TEM image, (**c**) N_2_ adsorption-desorption isotherms (inset: pore size distribution curve) and (**d**) Raman spectrum registered for the activated carbon (AC).

Physicochemical characterization of the superactivated carbon prepared by the *in-situ* polymerization, carbonization, and activation of melamine and terephthalaldehyde is included in Fig. 3. SEM images (Fig. 3a) show irregular-shaped carbon utricles with a size of ~50 nm and a very rough surface. High magnification TEM investigation (Fig. 3b) reveals the nanopores randomly distributed along with the sample. The N_2_ adsorption-desorption isotherm registered for this activated carbon exhibits a profile in between type I and IV with a distinguishable capillary condensation step in the relative pressure range of 0.3–0.6^19^. The abrupt increase of N_2_ absorption at low relative pressures is indicative of its highly microporous structure. Indeed, the specific surface area and pore volume calculated for this material are as high as 3180 m^2^ g^−1^ and 2.8 cm^3^ g^−1^, respectively. The pore size distribution calculated from the isotherm data (inset in Fig. 3c) shows the contribution of two pore systems with in the micro- and mesopore range, centered at *ca*. 1.0 nm and 2.3 nm, respectively. The ultra-large specific surface area combined with its hierarchical distribution of pore sizes is convenient for the physical adsorption of a large number of ions with a low resistance to diffusion, resulting ideal for its use as an EDLC electrode. The Raman spectrum in Fig. 3d displays the typical D and G bands at ~1350 cm^−1^ and ~1590 cm^−1^, respectively, pointing out that a significant amount of graphitic carbon is still present in the carbonaceous network despite the large concentration of defects and/or pores in sample^24^.

## Electrochemical characterization

**Figure 4:**
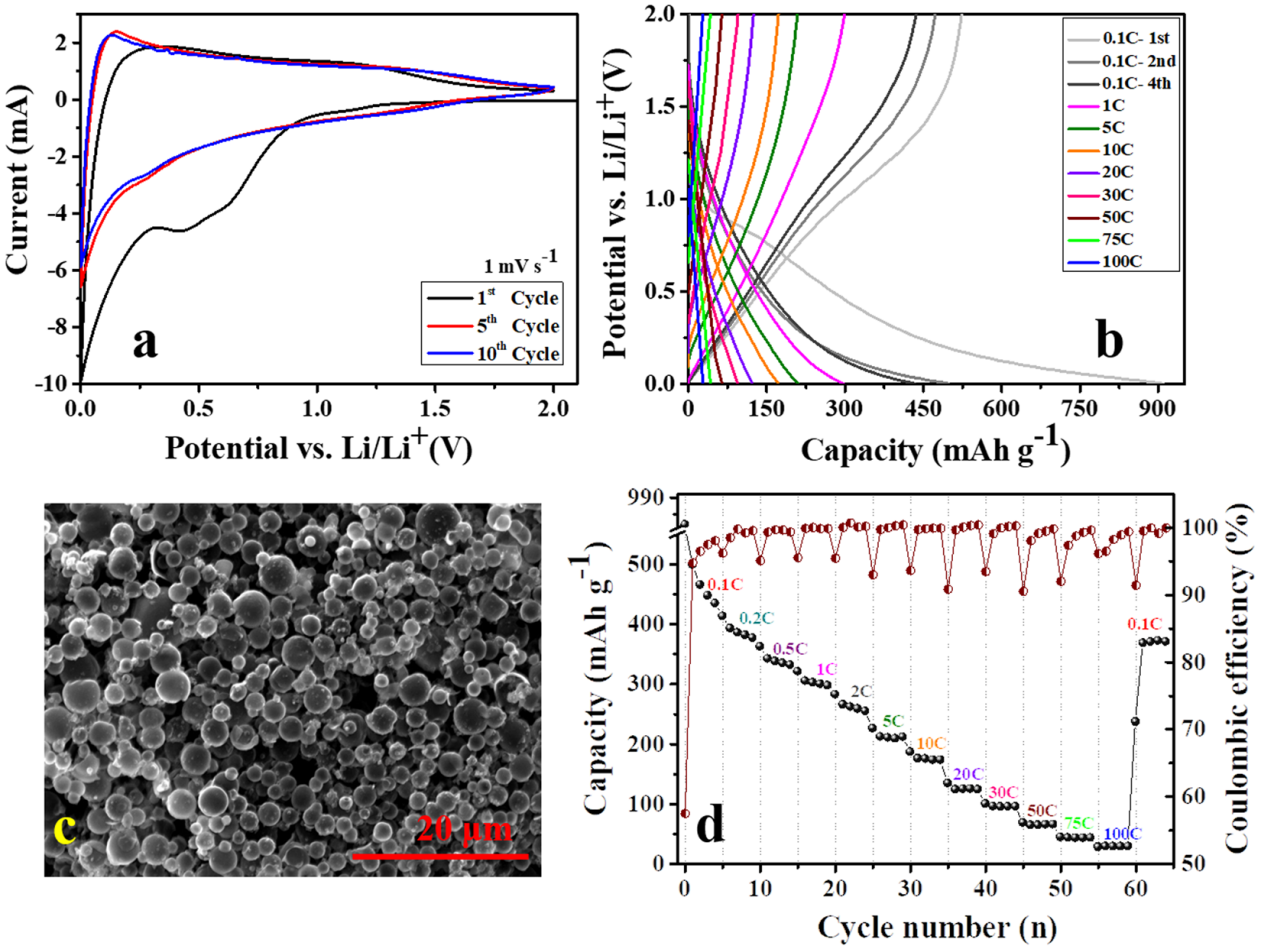
Electrochemical characterization of hollow carbon spheres as an anode in half-cell configuration tested between 0.002 and 2.0 V; (**a**) CVs *vs*. Li/Li^+^, (**b**) GC-GD *vs*. Li/Li^+^, (**c**) SEM image of HCS electrode surface and (**d**) rate capability and coulombic efficiency.

Both carbonaceous materials were electrochemically characterized individually. First, the performance of HCS as anode material was investigated in a half-cell configuration (T-type Swagelok) using Li foil as both the counter and the reference electrode. The cell was cycled within the potential range of 0.002–2.0 V *vs*. Li^+^/Li. Figure 4a illustrates the 1^st^, 5^th^ and the 10^th^ cyclic voltammograms (CVs) recorded at 1 mV s^−1^. It can be observed that most of the capacity is stored below 1.0 V. In the first CV, a broad reduction peak can be distinguished between ~1.0 to 0.3 V, which resembles the formation of a SEI layer due to the carbonate solvent decomposition^25^. The intercalation of Li^+^ into the HCS takes place between 0.3 and 0.01 V, while the deintercalation process shows a maximum current peak at 0.23 V. The Galvanostatic charge-discharge curves (GCD) performed between 2.0 V and 0.002 V at different current rates are shown in Fig. 4b. The first discharge at 0.1 C (C = 372 mA h g^−1^) from its open circuit potential shows two distinct plateaus at ~1.0 V and ~0.25 V corresponding to SEI formation and Li^+^ intercalation, which are in good agreement with the CV. The first discharge shows a very large specific capacity of *ca*. 910 mA h g^−1^, whereas the first charge shows a specific capacity of 523 mA h g^−1^ corresponding to an irreversible capacity loss of ~43%. Such high irreversible capacity loss measured in the first cycle is attributed not only to the formation of the SEI layer caused by the decomposition of carbonate electrolyte but also due to the irreversible reaction of Li^+^ with oxygen-containing functional groups present in the HCS^26^. It can be observed in the second and fourth charge-discharge curves that an additional charge storage occurs between 1.5 and 0.25 V in addition to the Li^+^ intercalation between 0.25 and 0.01 V. This explains the high specific discharge capacity values of 500 and 430 mA h g^−1^, respectively. This additional specific capacity values are attributed to the highly disordered nature of HCS carbon that promotes Li^+^ storage through other mechanisms such as excess bulk storage, storage in cavities and nanopores, interfacial/surface storage and the effect of heteroatoms, which gradually decreases during the subsequent cycles stabilizing after the fifth cycle^27^. The HCS anode showed excellent capacity retention at increased current rates (Fig. 4d). Thus, 173 mA h g^−1^ and 100 mA h g^−1^ were achieved at 10 C and 30 C (measured in the 5th cycle registered at each current rate), which corresponds to a retention of the initial capacity of ~40% and ~24%, respectively. Even after testing at the very high current rate of 100 C, 87% of the initial capacity was retrieved when the current rate was set again to 0.1 C. The SEM images registered for an anode containing HCS and the binder show that the carbon spheres are well dispersed, which ensures that lithium ions can easily access all the available microporous carbon surfaces (Fig. 4c). Additionally, the microstructure of the HCS is undoubtedly responsible for such advanced rate performance. Both the interparticle space and the spherical voids in the core of the HCSs act as ion-buffering reservoirs, which shorten the diffusion path towards the thin microporous carbon shell^16,17^. It is also noteworthy that although the CE in the first cycle was merely 57%, it quickly raised up to 95% in the second cycle and stabilized at a value of 98% in the subsequent cycles even at high current rates. Such high CE indicates that this carbon architecture is very capable of enduring the mechanical stress induced at harsh current rates.

**Figure 5:**
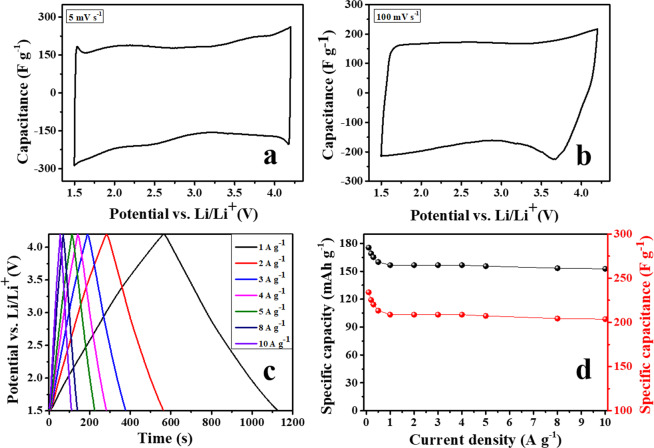
Electrochemical characterization of AC cathode in half-cell configuration tested between 1.5 to 4.2 V; (**a**) & (**b**) CVs at low and high scan rate of 5 mV s^−1^ and 100 mV s^−1^ (*vs*. Li/Li^+^), (**c**) GC-GDs *vs*. Li/Li^+^, and (**d**) capacity *vs*. current density (rate performance).

The capacitive performance of the superactivated carbon was evaluated in the potential range of 1.5–4.2 V *vs*. Li^+^/Li using LiPF_6_ in 1:1 (EC:DMC) as the electrolyte. Figure 5a,b include the CV curves registered at 5 and 100 mV s^−1^. At the lowest scan rate, the plot is square-shaped and very symmetric, evidencing the capacitive behavior of the material. Generally, the open circuit potential of activated carbon cathodes falls in between the potential range of 3.0–3.1 V *vs*. Li^+^/Li in a Li-ion electrolyte. Therefore, the electrical double layer stores ions of opposite charge depending on the potential range, *i.e*. it adsorbs ${{\rm{PF}}}_{6}^{-}$ anions from 3 V to 4.2 V and Li^+^ cations from 3 V to 1.5 V. Even at the high sweep rate of 100 mV s^−1^ the plot shows the characteristic rectangular-shaped profile, pointing out the fast and effective polarization undergone due to the charge separation at the electrode/electrolyte interface. Figure 5c shows the GC-GD profiles of the AC cathode at different current densities. The symmetric triangular-shaped GC-GD curves showing almost 100% of CE confirm the purely capacitive behavior of the superactivated carbon. Interestingly, this AC achieved a specific capacitance of 208 F g^−1^ at 1 A g^−1^, and retained 203 F g^−1^ at a high discharge rate of 10 A g^−1^ (Fig. 5d). Such good capacitance retention is favored by the extremely high specific surface area of the AC combined with its hierarchical and interconnected porous network, which allows the unimpeded diffusion of electrolyte ions onto the active carbon surface^19,20^. The excellent rate capability observed together with the absence of ohmic drop at the beginning of the discharge branches point out this activated carbon as a promising positive electrode material for LIC systems.

**Figure 6:**
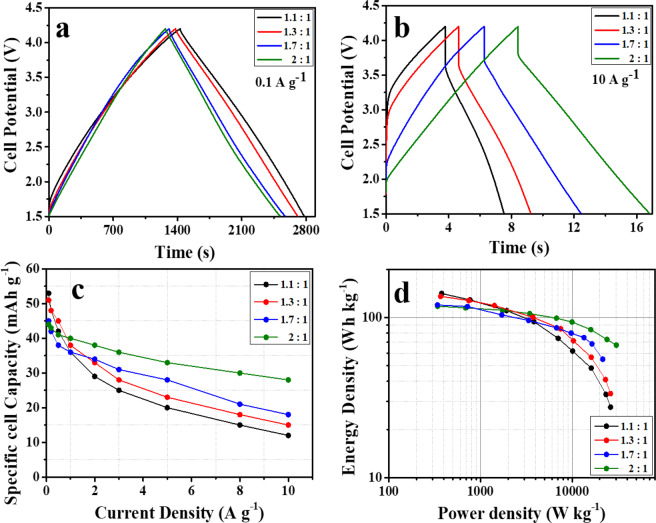
Comparative electrochemical characterization of HCS//AC full cells with different electrode mass ratios: GC/GDs at a current density of (**a**) 0.1 A g^−1^ and (**b**) 10 A g^−1^, (**c**) rate capability and (**d**) Ragone plot.

In view of the good performances exhibited by both carbonaceous materials, LIC full cells were assembled using HCS and AC as anode and cathode electrodes, respectively. As a rule of thumb, in asymmetric capacitor configuration there should be charge balance between anode and cathode based on the specific capacity and potential window^28^. However, this rule does not always result in optimum performance in the case of a Li-ion capacitor. This is because there is stark difference between the kinetics of faradaic lithiation in anode and non-faradaic ${{\rm{PF}}}_{6}^{-}$ adsorption on cathode. This difference results in contrasting specific capacity performance of anode and cathode at low and high current density (Figure S3 of Supplementary information), which make it almost impossible to estimate the charge balance effectively only by considering the specific capacity at low current. Therefore, to evaluate and optimize the electrochemical performance of the full cell as well as to achieve best performance in terms of specific capacity, cycling stability and safety, a variation of the electrode mass ratio was investigated. Thus, four LIC cells were assembled using anode/cathode electrode mass ratios of 1.1, 1.3, 1.7 and 2.0. In Fig. 6a,b have compared the GC-GD curves recorded for these four LIC cells at 0.1 and 10 A g^−1^, respectively, in the 1.5–4.2 V potential range. At the lowest current density, a progressive decrease of the discharge time was observed when the mass ratio was increased from 1.1:1 to 2:1. This trend is inverted when the current density is increased to 10 A g^−1^. At this current rate, a prominent decrease in the ohmic drop combined with an increase in the discharge time (almost two-fold higher) is observed when the mass ratio is increased from 1.1 to 2. This difference in mass variation performance is also clearly exhibited from Ohmic drop *vs*. current density plots of all LIC cells is shown in Figure S1 of the Supplementary Information. The better electrochemical performance of the high mass ratio LIC cell seems to be the deeper utilization (larger operating voltage) of the EDLC electrode during the fast anionic adsorption-desorption process. Figure 6c shows the evolution of the specific capacity with the current density for the LIC cells using different electrode mass ratios. It can be observed that at current densities below 1 A g^−1^ the LIC cell with the lower mass ratio (1.1:1) shows the highest specific capacity, whereas the LIC cell assembled using the 2:1 electrode mass ratio shows the best rate capability and the largest value of specific capacity at high current rates, achieving 28 mA h g^−1^ at a current density of 10 A g^−1^. The same trend with respect to the mass variation is also noticed in case of specific cell capacitance (F g^−1^) values, which are included in the Figure S2 (Supplementary Information). Figure 6d represents the comparative Ragone plots calculated for the LICs with different electrode mass ratios. At the lowest current density, the 1.1:1 cell achieved an energy density of 141 Wh kg^−1^, and this value slightly decreased with the increase of the mass ratio down to the 117 Wh kg^−1^ reached by the 2:1 cell. With the increase of the applied current, the differences between the different cells become more noticeable. Indeed, at the highest current density (8 seconds of discharge) the LIC with the highest loading in the negative electrode obtained an energy density as high as 68 Wh kg^−1^ at a power density of 30 kW kg^−1^.

**Figure 7:**
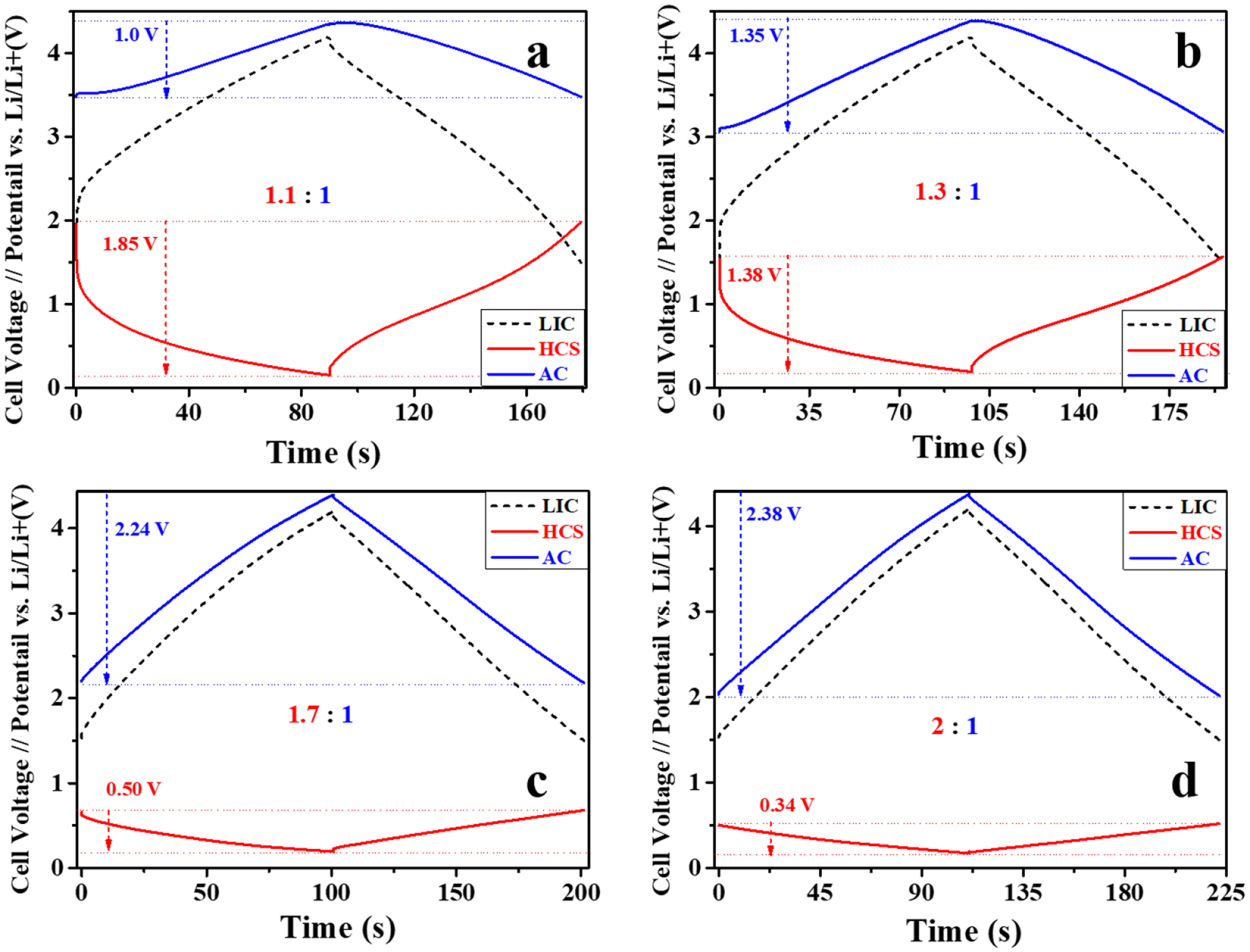
Comparative galvanostatic charge-discharge profiles of each electrode in LICs with different electrode mass ratios at a current density of 1 A g^−1^. The cells were run in the 1.5–4.2 V potential range.

In order to get deeper insights into the electrochemical performance of the LICs, the performance of each electrode was monitored. The GC-GD profiles registered for the AC cathodes and the HCS anodes (plotted *vs*. Li/Li^+^) at a current density of 1 A g^−1^ are shown in Fig. 7a–d. In the 1.1:1 LIC cell (Fig. 7a) the anode potential swing is significantly high (~1.85 V), which evidences a high utilization of the anode for the Li^+^ intercalation-deintercalation process. Therefore, this configuration allows extraction of the highest amount of charge stored thus delivering the highest specific capacity. However, since the anodic process is kinetically much slower than the adsorption-desorption of ${{\rm{PF}}}_{6}^{-}$ occurring in the positive electrode, this configuration limits the charge extraction at high current densities. This anode potential swing is gradually decreased from ~1.38 to ~0.34 V, as it can be observed from Fig. 7b–d, with an increase in anode/cathode mass ratio. This decrease results in a less utilization of the anode but results in less particle volume expansion, electrolyte decomposition and lithium consumption. On its behalf, with the increase in anode/cathode mass ratio, the potential swing in the cathode increased significantly from 1.0 V (1.1:1 cell) to 2.38 V (2:1 cell) thus gradually enhancing the cathode capacity. Therefore, the 2:1 electrode mass ratio guarantees the best rate capability of the cell taking advantage of a higher utilization of the porous electrode surface. On the other hand, the lower polarization registered in the anode limits the operative capacity of the anode thus results in less specific capacity of full cell. However, concurrently this lower anode polarization avoids the chances of lithium plating, which is beneficial in terms of safety as well as durability.

**Figure 8:**
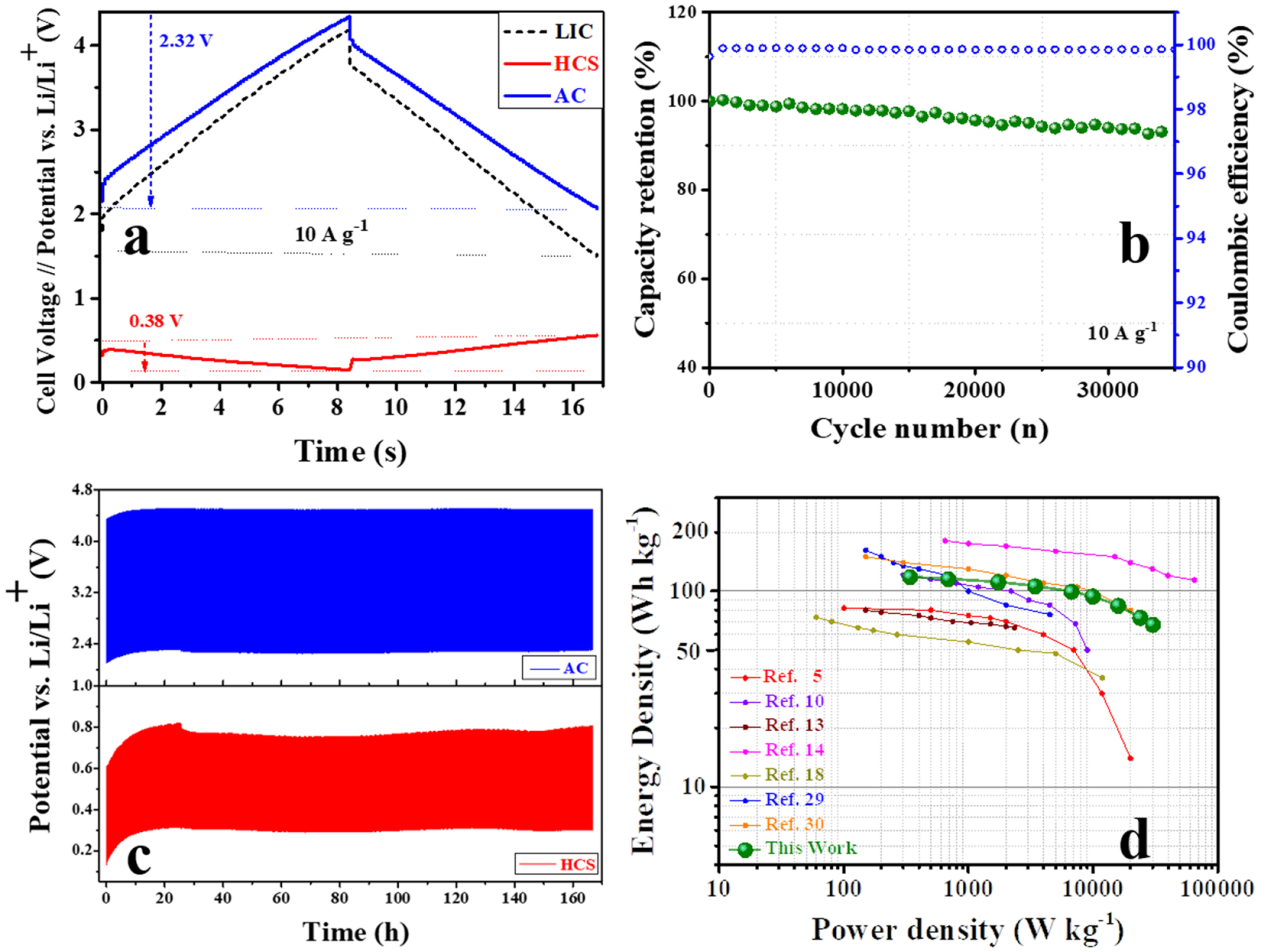
Electrochemical performance of the 2:1 LIC: (**a**) GD-GC profile of each electrode and the full cell potential window at a current density of 10 A g^−1^; (**b**) cycling stability; (**c**) potential swings of each electrode during the cycling test; (**d**) comparative Ragone plots of our 2:1 LIC and other representative LICs reported in the literature.

Figure 8a and Figure S4 in supplementary information show the GC-GD plots of each electrode as well as the corresponding LIC for all cell combinations at a current density of 10 A g^−1^. These figures almost mirror the observation revealed from Fig. 7a–d with additional evidence. Figure 8a reveals that the 2:1 LIC cell is still fully operative within the 1.5–4.2 V potential range even at such a high current rate. Moreover, the anode potential swing is still limited to ~0.38 V. However, the 1.1:1 LIC cell (Figure S4a) shows a significant increase in anode potential swing up to ~2.26 V. This results in severe Li^+^ plating on the anode (yellow color marked area in Figure S4), which not only degrades the cell performance but also aggravates safety issues. The other two mass ratio LIC cells show a gradual decrease in the anode potential swing, thus enhancing the power performance. From all these GC-GD results, it was found that the 2:1 LIC cell shows the best performance in terms of both specific capacity and rate capability. This is due to a steady cathode potential window combined with the contended use of the anode (its CE is very close to 100%), which allows the stable performance of the full cell. So, this 2:1 LIC cell is selected to investigate the long-term stability by performing GC-CD cycles at a current density of 10 A g^−1^. The resultant cyclic stability plot in Fig. 8b shows that this LIC cell exhibited an outstanding cycling performance, retaining 98.7% of its initial capacity after 10,000 cycles, and 92% after 35,000 cycles. The potential swing of each electrode during the cycling test is plotted in Fig. 8c. As can be seen, the cathodic potential window shows a small upward shifting, but remains steady during the whole test, which validates the stable performance of the full cell.

For the sake of comparison, Fig. 8d collects the Ragone plot of our optimized LIC as well as those of other representative LIC systems recently reported. It is worth to highlight the excellent energy density measured for our selected LIC, especially in the high-power region in which most of the previously reported LICs suffer from an abrupt decay of their energy densities^5,10,13,14,18,29,30^.

## Conclusions

Micro-sized hollow carbon spheres have been synthesized by an easy procedure. This material exhibits improved performance in the lithium insertion-extraction process especially at very high current rates, which point it as a promising candidate for its use as the negative electrode in lithium-ion capacitors. LICs were assembled by coupling this micro-structured hollow carbon spheres *versus* a superactivated micro-mesoporous carbon using different electrode mass ratios. The hollow carbon spheres are able to resist the volume changes during repetitive lithiation-delithiation cycles, while the hierarchical porosity of the superactivated carbon offering very low resistance to ion diffusion assured a good response at high current rates. It was found that the best negative/positive electrode mass ratio in this LIC system is 2:1, at which the cell delivers a maximum gravimetric energy density of 117 Wh kg^−1^ at 0.34 kW kg^−1^ and still 68 Wh kg^−1^ at an extreme power density of 30 kW kg^−1^. The robustness of the LIC was confirmed by its remarkable long-term stability over 35000 cycles with only 8% of capacity decay registered. This outstanding performance makes our proposed LIC a promising energy storage system standing out among its peers.

## Experimental Section

### Synthesis

The hollow carbon spheres (HCS) were synthesized by the carbonization of polymeric hollow spheres under a dynamic inert atmosphere. For the synthesis of the co-polymeric hollow nanospheres, 0.08 g of Triton-X-100 was dispersed in 50 ml deionized water, and then 0.456 ml of aniline and 0.346 ml of pyrrole were added to the mixture under continuous stirring that was kept until complete dissolution. Then, the solution was kept under continuous stirring in an ice bath to maintain a temperature of 3–5 °C. For the oxidative polymerization, aqueous ammonium persulfate (0.8 g was dissolved in 1 ml DI water) was precooled at 3–5 °C and added to the above solution. The mixture was stirred for a few minutes and the resulting solution was kept in the refrigerator for 24 h at 4 °C. Finally, the obtained dark greenish polymer precipitate was collected by centrifugation and washed with DI water several times. The product was freeze-dried to maintain the microscopic structure of the polymeric hollow spheres and then carbonized at 800 °C in Ar atmosphere for 2 h using a heating rate of 3 °C min^−1^.

The superactivated carbon (AC) was synthesized following the synthetic route described in detail in our previous report^19^. Briefly, 1.24 g of melamine, 1.36 g of terephthaladehyde and 5.0 g of KOH were grounded using an agate mortar and the mixture was carbonized under Ar atmosphere. The temperature was first raised up to 250 °C for 3 h and then increased to 800 °C for 1 h using heating ramps of 1 °C min^−1^. (CAUTION: certain amount of potassium cyanide may be formed during the carbonization process, so carbon should be carefully manipulated, and the wastes treated accordingly). Then the final product was washed several times with 3 M HCl and DI water followed by drying at 120 °C in an oven.

### Physicochemical characterizations

X-ray diffraction (XRD) patterns of the synthesized powdered samples were recorded on a Bruker D8 X-ray diffractometer and the data were attained at 40 kV and 30 mA using CuKα radiation over 2θ within the range from 5 to 90° at steps of 0.02° with a residence time of 5 seconds. Raman spectra data were collected using a Renishaw spectrometer (Nanonics Multiview 2000) which was operated with an excitation wavelength of 532 nm under an Ar ion laser with an exposition time of 10 seconds. The nanostructure of the synthesized samples was investigated on a Scanning electron microscope (SEM) in a field emission Quanta 200 FEG microscope. Tecnai G2 transmission electron microscope (TEM, FEI) was used for the microstructural characterization. For TEM analysis, samples were homogeneously dispersed in 1 ml ethanol for 10–15 min by ultrasonication. After that, a few drops of the solution were cast on a Cu grid decorated with holey carbon films. N_2_ adsorption-desorption experiments were carried out at −196 °C using an ASAP 2020 instrument from Micromeritics. The values of specific surface area were calculated using the Brunauer, Emmett, and Teller (BET) equation within a relative pressure range of 0.05–0.2. The total pore volume (V_T_) was calculated by the amount of nitrogen adsorbed at р/p_o_ = 0.95. Pore size distributions (PSD) were evaluated based on the N_2_ adsorption branch data by using the two-dimensional nonlocal density functional theory (2D-NLDFT). The nitrogen content in the HCS carbon was determined by inductively coupled plasma mass spectrometry (ICP-MS).

### Electrode preparation, cell assembly, and electrochemical characterization techniques

The negative electrode slurry was prepared by mixing 90 wt% of hollow spherical carbon (HCS) with 5 wt% Super-C C65 carbon black (Imerys Graphite & Carbon, Willebroek, Belgium) and 5% polyvinylidene fluoride (PVdF) in N-methyl-2-pyrrolidone (NMP). The components were mixed under vigorous stirring for at least 1 h using a magnetic stirrer. The obtained HCS-based slurry was coated onto a copper foil current collector. For the positive electrode slurry, the activated carbon, Super-C C65, and PVdF were mixed in a weight mass ratio of 90:5:5 in NMP solution under continuous stirring for 1 h and then the AC-based slurry was laminated onto an aluminum foil. Laminates were placed immediately into a vacuum oven for drying at 80 °C for 12 h under constant vacuum. The mass loading of the positive electrode was of 1–1.3 mg cm^−2^ while the loading in the negative electrode ranged from 1.4 to 2.6 mg cm^−2^. The electrochemical characterization of the anode was evaluated in a three-electrode configuration using an airtight Swagelok T-cell. Metallic Li was used as both the counter and the reference electrode, and the anode was cycled within the potential range of 0.002 V to 2 V. The same cell assembly procedure was followed to perform the electrochemical characterization of the cathode within the 1.5–4.2 V potential range.

Lithium hybrid supercapacitor full cells (HCS//AC) were assembled using four different negative-to-positive electrode mass ratios: (1.1:1), (1.3:1), (1.7:1) and (2:1). A three-electrode configuration (Swagelok T-cell) with a metallic Li reference was chosen in order to record the individual electrode potential changes. Stainless steel current collectors and a porous glass fiber separator (Whatman GFB) were used and the electrolyte used was 1 M LiPF_6_ in EC:DMC (1:1). Before testing, the negative and positive electrodes were preconditioned to maximize the output voltage. Thus, the HCS electrode was cycled at least five times between 0.002 and 2 V *vs*. Li/Li^+^ at 0.1 C rate to form a solid electrolyte interphase (SEI) and supply enough lithium to compensate the initial irreversible cycles. After that, a cut-off potential of 0.2 V *vs*. Li/Li^+^ was set to evade any chances of lithium plating. The AC electrode was also charged up to a cut-off potential of 4.2 V *vs*. Li/Li^+^. After this pre-lithiation process, the LICs full cells were built for their extensive electrochemical characterization. Cyclic voltammetry (CV), and Galvanostatic charge-discharge (GC-GD) measurements were performed using a multichannel VMP3 generator (Biologic, France).

### Calculations

The gravimetric specific capacitance *C*_*D*_ (F g^−1^) of LIC was calculated from the discharge curve in GC-GD measurements using the following equation:

1 $${{\boldsymbol{C}}}_{{\boldsymbol{D}}}=\frac{I{t}_{D}}{{m}_{el}{U}_{D}}$$

where *I* is the constant current applied, *t*_*D*_ is the time spent during discharge, *m*_*el*_ is the active mass of both electrodes and *U*_*D*_ is the voltage difference during discharge.

The discharge specific capacity *Q*_*D*_ (mA h g^−1^) of the cell was calculated according to the following equation:

2 $${{\boldsymbol{Q}}}_{{\boldsymbol{D}}}=\frac{I{t}_{D}}{{m}_{el}3.6}$$

where *I* is the constant current (A); *t*_*D*_ is the time spent during discharge (s) and *m*_*el*_ the mass (g) of both electrodes.

The coulombic efficiency (*η*_*t*_) was calculated by the ratio between the discharge and charge times.

3 $${{\boldsymbol{\eta }}}_{{\boldsymbol{t}}}=\frac{{t}_{int,D}}{{t}_{int,C}}$$

Since the hybrid system exhibits battery-like non-linear GC/GD curves, the discharge specific energy density (*ED*, W h kg^−1^) was determined by integrating the area under the GD curve^31^.

4 $${\boldsymbol{ED}}=0.277J{\int }_{t({U}_{max})}^{t({U}_{min})}U(t)dt$$

where *t(U*_*min*_) and *t(U*_*max*_) the time corresponding to the minimum and maximum voltage in the discharge portion of a galvanostatic cycle (s); *U(t)* the instant voltage (V) and *J* is the current density.

The power density *PD* (W kg^−1^) was calculated according to:

5 $${\boldsymbol{PD}}=ED\times 3600/[{t}_{D}({U}_{max})-{t}_{D}({U}_{min})]$$

The values of energy and power density obtained at different specific currents were used to draw Ragone Plot^31^.

## Supplementary information

Supplementary Information.
