# Publisher Correction: Fabrication of high-performance dual carbon Li-ion hybrid capacitor: mass balancing approach to improve the energy-power density and cycle life

**DOI:** 10.1038/s41598-020-69896-x

**Published:** 2020-10-19

**Authors:** Tandra Panja, Jon Ajuria, Noel Díez, Dhrubajyoti Bhattacharjya, Eider Goikolea, Daniel Carriazo

**Affiliations:** 1 Centre for Cooperative Research on Alternative Energies (CIC energiGUNE), Basque Research and Technology Alliance (BRTA), Alava Technology Park, Albert Einstein 48, 01510 Vitoria-Gasteiz, Spain; 2 Universidad del País Vasco, UPV/EHU, 48080 Bilbao, Spain; 3 Instituto de Ciencia y Tecnología del Carbono, INCAR-CSIC, Francisco Pintado Fe, 26, 33011 Oviedo, Spain; 4 IKERBASQUE, Basque Foundation for Science, 48013 Bilbao, Spain

Correction to: *Scientific Reports* 10.1038/s41598-020-67216-x, published online 02 July 2020

In the Supplementary Information file originally published with this Article, Supplementary Figures 3 and 4 were omitted. These errors have been corrected in the Supplementary Information that now accompanies the Article.

