# Supplementary material for: Fabrication of high-performance dual carbon Li-ion hybrid capacitor: mass balancing approach to improve the energy-power density and cycle life
Source: Sci Rep. 2020 Jul 2;10:10842. doi: 10.1038/s41598-020-67216-x (PMC7331633; doi:10.1038/s41598-020-67216-x)
Supplement: Supplementary file 1 — Supplementary Information. [file 41598_2020_67216_MOESM1_ESM.docx]

**Supporting Information**

Fabrication of high-performance dual carbon Li-ion hybrid capacitor: mass balancing approach to improve the energy-power density and cycle life

*Tandra Panja,^a, b^ Jon Ajuria,^a^ Noel Díez,^c^ Dhrubajyoti Bhattacharjya,^a^ Eider Goikolea,^b^ and Daniel Carriazo^*a, d^*

^a^ Centre for Cooperative Research on Alternative Energies (CIC energiGUNE), Basque Research and Technology Alliance (BRTA), Alava Technology Park, Albert Einstein 48, 01510 Vitoria-Gasteiz, Spain.

^b^ Universidad del País Vasco, UPV/EHU, 48080 Bilbao, Spain

^c^ Instituto Nacional del Carbón-INCAR (CSIC), 33080 Oviedo, Spain

^d^ IKERBASQUE, Basque Foundation for Science, 48013 Bilbao, Spain


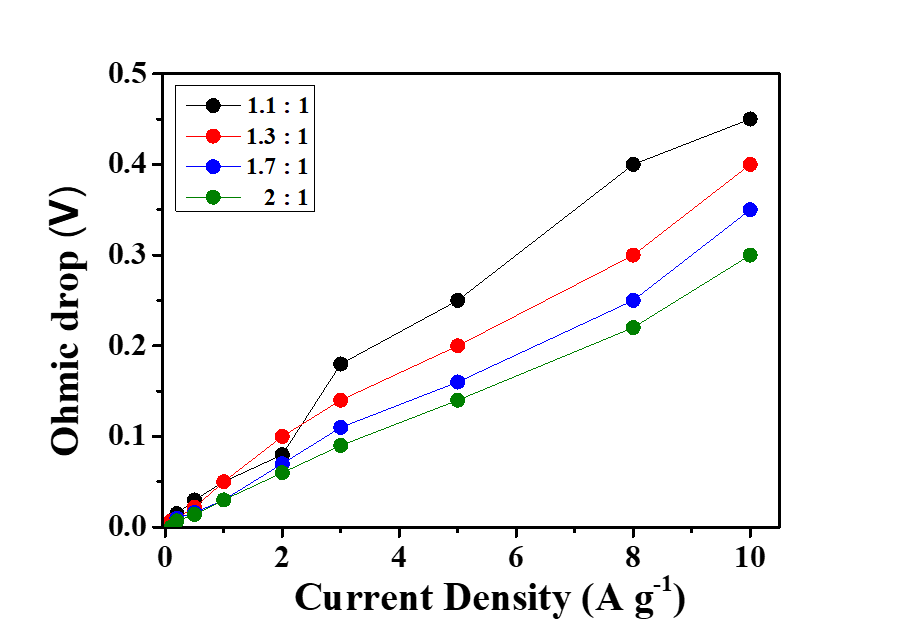


**Figure S1.** Comparative ohmic drop values with respect to different current densities for the different LIC devices assembled using indicated positive to negative mass balances.


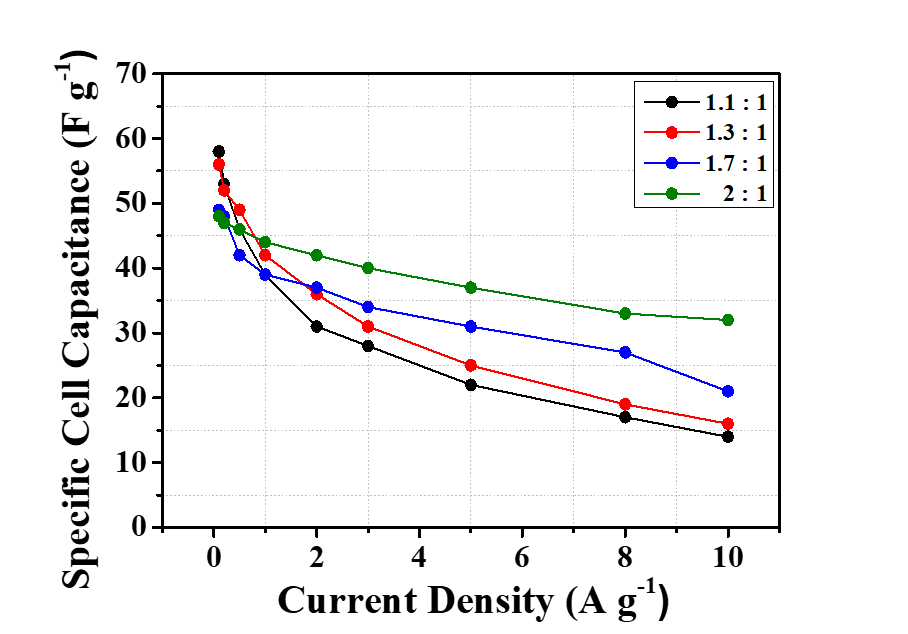


**Figure S2.** Specific cell capacitance evolution for the different LIC devices assembled

using labeled negative to positive mass ratios.

The specific cell capacitance (***C_D,cell_***) of the full cell was calculated according by the

following equation:

$$\boldsymbol{C}_{\boldsymbol{D,cell}}\boldsymbol{=}\frac{\boldsymbol{2}}{\boldsymbol{U}_{\boldsymbol{cell}}^{\boldsymbol{2}}}\boldsymbol{ED}$$

Where, discharge specific energy density ***ED*** (Wh kg^-1^) and ***U_cell_*** the voltage in the

discharge curve of a galvanostatic cycle, V.
